# Cycles of external dependency drive evolution of avian carotenoid networks

**DOI:** 10.1038/s41467-019-09579-y

**Published:** 2019-04-08

**Authors:** Alexander V. Badyaev, Alexander B. Posner, Erin S. Morrison, Dawn M. Higginson

**Affiliations:** 1 Department of Ecology & Evolutionary Biology, University of Arizona, Tucson, AZ 85721 USA; 2 Department of Epidemiology & Biostatistics, School of Public Health, University of California, Berkeley, CA 94720 USA; 3 Sackler Institute for Comparative Genomics, American Museum of Natural History, New York, NY 10024 USA

## Abstract

All organisms depend on input of exogenous compounds that cannot be internally produced. Gain and loss of such dependencies structure ecological communities and drive species’ evolution, yet the evolution of mechanisms that accommodate these variable dependencies remain elusive. Here, we show that historical cycles of gains and losses of external dependencies in avian carotenoid-producing networks are linked to their evolutionary diversification. This occurs because internalization of metabolic controls—produced when gains in redundancy of dietary inputs coincide with increased branching of their derived products—enables rapid and sustainable exploration of an existing network by shielding it from environmental fluctuations in inputs. Correspondingly, loss of internal controls constrains evolution to the rate of the gains and losses of dietary precursors. Because internalization of a network’s controls necessarily bridges diet-specific enzymatic modules within a network, it structurally links local adaptation and continuous evolution even for traits fully dependent on contingent external inputs.

## Abstract (web summary)

The mechanisms that accommodate variable external dependencies in evolution are not clear. Here, the authors show that switches between external and internal metabolic controls of carotenoid-producing networks in birds are linked to shifts in evolutionary rates, with internalization of control resulting in bursts of evolutionary diversification.

## Introduction

Evolution proceeds by forming continuous lineages from past functional solutions of ancestors to the features of extant forms. In this historical process, as species evolve adaptations and form associations with other species, they inevitably gain and lose their dependence on inputs from the external environments and other species^1–3^. Gain and loss of such external dependencies can structure ecological communities^4^ and alter metabolic^5^ and genomic^6,7^ architecture, yet the mechanisms allowing modifications of these dependencies remain elusive. Particularly illuminating in the search for such mechanisms is a proposition that evolution requires historical continuity among the elements of contemporary phenotypes (e.g., genes or proteins) on some level of organization^8,9^. Evolving lineages form physical, developmental and functional associations of these elements and changing boundaries of these highly contingent phenotypic associations^10^ result in evolutionary divergence and transient ecological linkages^1,5,11–15^. Although empirical and conceptual studies have established the centrality of this principle in explaining biological diversity^16–18^, it remains unclear how to reconcile historical stability of these linkages with their frequent gain and loss associated with involvement into contemporary innovations. In other words, how do transient functional roles of existing elements coexist with the historical maintenance of their links to other elements^8^?

This problem is captured by the concept of network controllability which concerns the ability to change a network configuration while maintaining its functioning^19,20^. The network nodes that are influential in this process—the control nodes—acquire this status because of their topological position or connectivity (see Methods). To the extent that these properties reflect a network’s current function, the propensity of a node to control the network can be gained or lost, thereby providing a mechanistic link between a current adaptation and evolutionary change^21^. Indeed, the distribution of control nodes on a biological network is thought to emerge as a compromise between the competing demands of robustness and evolvability of functional states^22–24^ and may constitute the unifying principle of their organization^25,26^. Yet, the acquisition and persistence of controlling propensities on evolving biological networks have not been studied, although ontogenetic changes in controllability of biological networks are beginning to be examined^24,27^.

**Fig. 1:**
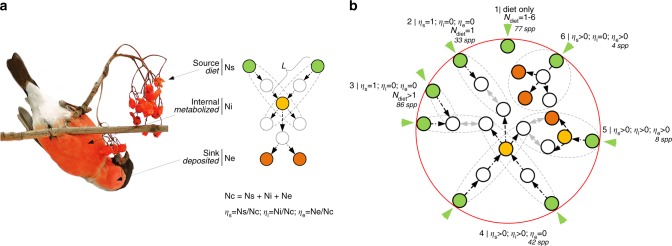
Functional correspondence of controlling structures in avian carotenoid networks. **a** Control nodes classified into source (Ns), internal (Ni), and sink (Ne) controls based on the asymmetry of their incoming and outgoing reactions (arrows) correspond to dietary carotenoids (green circles), metabolized carotenoids (yellow), and terminal branching leading to plumage-bound carotenoids (red), respectively (see Methods section). Dashed lines outline parts of biochemical modules linking dietary and derived compounds, *L* is the length of path (in reactions) before a pathway starting from one dietary carotenoid merges with a pathway starting from a different dietary carotenoid at the degenerate node (yellow). A minimum number of controls (Nc) required to fully control a network is the sum of the number of source, internal and peripheral controls. Proportion of Nc due to sources (*η*_s_), internal branching (*η*_i_), and peripheral sinks (*η*_e_) is the control profile of a network^29^. **b** Networks of study species, schematically grouped by their control profiles. Green triangles are dietary inputs, gray double-headed arrows mapped on the combined avian enzymatic network of carotenoids (enclosing red circle) illustrate potential transitions between control categories. Species in category 1 deposit only dietary carotenoids into their plumage. Networks in categories 2 and 3 are fully controlled by sources (*η*_s_ = 1). In categories 4 and 6 full control of networks requires one additional control due to either internal (*η*_i_ > 0) or sink (*η*_e_ > 0) controls, correspondingly. The networks in category 5 require all three types of controls

Evolution of enzymatic networks that produce carotenoid-based ornamentation in birds is particularly suitable for such a study because the position and connectivity of control nodes in these networks correspond to their biological functions (Fig. 1a). Birds cannot produce carotenoids from non-carotenoids and thus consume exogenous dietary carotenoids and metabolically convert them into derived carotenoids before depositing them in their plumage^28^. In these networks, nodes are carotenoid compounds and arrows (edges) show enzymatic conversion. Inherent asymmetry in in- and outbound node connectivity in avian carotenoid networks in relation to their biological roles (Fig. 1a), enables classification of dietary carotenoids as source controls, derived carotenoids that have more outgoing than incoming reactions (i.e., points of metabolic expansion) as internal controls, and some peripheral branching associated with deposition of carotenoids into the integument as the sink controls^29^ (Fig. 1a, see Methods for derivation). With this classification, historical changes in the proportion of controls types—network’s control profile^29^—provide insight into whether evolution of biochemical basis of avian carotenoid coloration is associated with historical changes in proportion of source controls, caused by changes in diversity of dietary carotenoids, changes in proportion of internal controls, caused by the enzymatic evolution, or changes in proportion of sink controls, caused by the diversification of feather keratin-carotenoid associations. Thus, the study of historical changes in control profiles enables insights into the mechanistic bases by which changes in external dependencies of organisms affect their evolution—a long standing goal in evolutionary biology^1,4,30–32^.

Here we report that the evolution of biochemical networks that produce diet-dependent coloration in birds is driven by historical transitions between external and internal metabolic controls of these networks. We show that internalization of control enables rapid and sustainable exploration of an enzymatic network and is associated with bursts of evolutionary diversification. We further uncover historical cycles of transition between network control types and show how they are determined by juxtaposition of a species’ diversity of dietary carotenoids and distribution of underlying network connectivity. We show how historical transitions in controllability of carotenoid networks mechanistically link diversification and stasis in avian carotenoid evolution.

## Results

### Extent of internal control in avian carotenoid metabolism

**Fig. 2:**
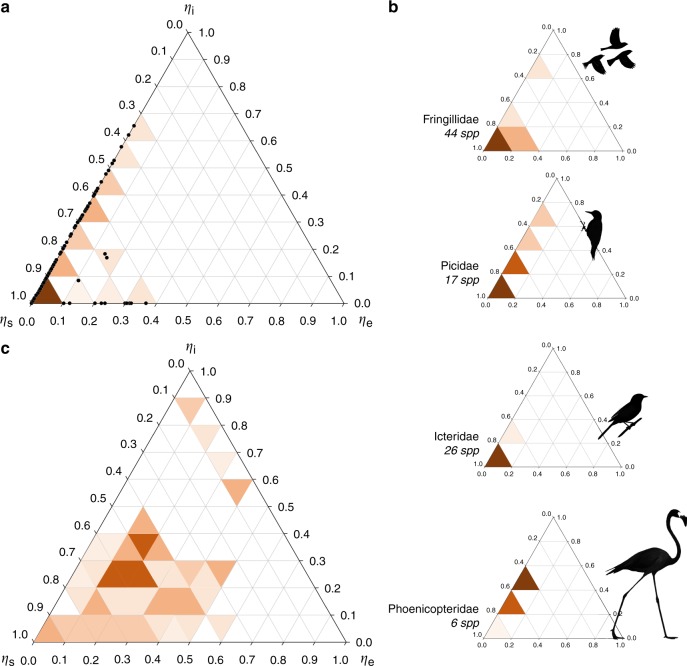
Distinct control profiles of avian carotenoid networks and their randomized counterparts. **a** Heatmap of control profiles for networks of 250 bird species, projected to a ternary plot, reveals source-dominance, a significant extent of internal control, a deficit of sink controls, and a lack of mixed controls. Shades indicate density of networks (in 10% increments) with control profiles in that ternary space, data points are individual species. **b** Clustering of network control profiles of selected clades of birds with significant internal controls (Supplementary Fig. 1 shows additional clades). **c** ER randomization reveals that avian networks avoid mixed controls, have greater redundancy in source controls, and accumulate more enzymatic expansions that need internal controls than their randomized counterparts of the same size and complexity (Supplementary Fig. 2). Drawing sources are in Supplementary Fig. 1

We first derived the control profiles for carotenoid-producing networks of 250 bird species (Fig. 2, Supplementary Data 1). Although the profiles were dominated by sources, as expected for traits dependent on dietary inputs, we observed a surprising extent of internal control in networks of many lineages (Fig. 2b). In a number of taxa, networks were wired in such a way that the control exerted by the enzymatic branching (internal control) exceeds that caused by consumption of dietary carotenoids (source controls) (Supplementary Fig. 1).

**Fig. 3:**
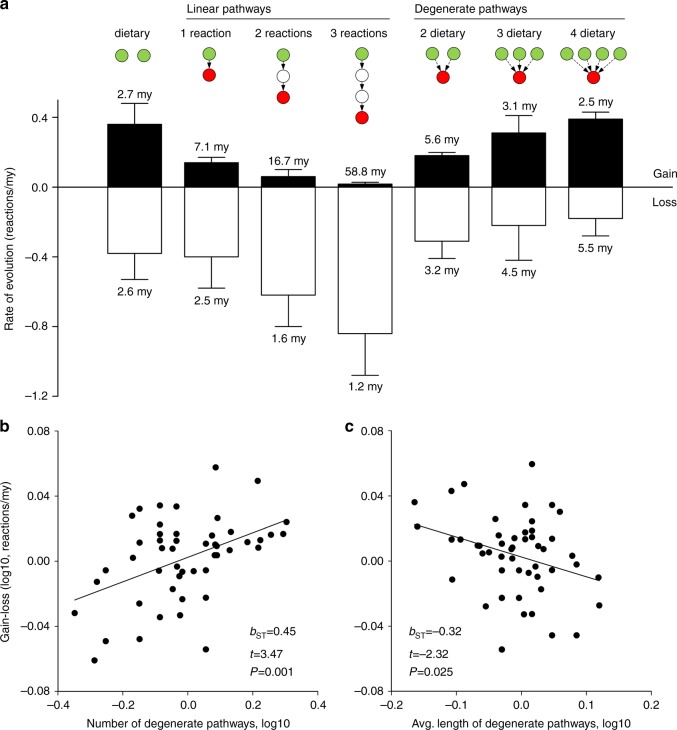
Metabolic degeneracy sustains evolution of avian carotenoid networks. **a** Node occupancy rate (gains vs losses of nodes, in millions of years [my] ± 1 s.e.m.) in relation to distance (in reactions) from dietary inputs in linear (left group) and degenerate (right group) pathways. Average frequency of a loss of a dietary carotenoid (leftmost bar) sets the lowest limit for elongation of enzymatic pathways—sustained elongation is only possible when a new derived node is occupied at higher rate. Only degenerate pathways supported by >2 dietary carotenoids enable sustained growth of network. Under all other scenarios losses dominate, leading to network shrinkage or stasis. **b**, **c** Derived compounds closer to dietary carotenoids and those sustained by more paths are occupied more frequently in avian evolution—they are encountered faster and take longer to lose. Partial regression plots of a likelihood of gain minus the likelihood of a loss of a node regressed on **b** the number of degenerate pathways reaching this node, and **c** the average length of these pathways (Supplementary Data 6)

To separate biologically significant features of observed control profiles from geometric consequences of network size and connectivity, we randomized each species’ network with Erdös–Rényi (ER) randomization of degree distribution (Fig. 2c). ER randomization revealed that real avian carotenoid networks required more controls than a randomized network of the same size and complexity (Supplementary Fig. 2) and identified three key features of real carotenoid networks—(1) redundancy in source controls was associated with accumulation of metabolic expansions that required internal controls, (2) sink controls were rare, and (3) networks were dominated by only one control type at any given time (i.e., control profiles clustered along periphery of control space, Fig. 2c; Supplementary Fig. 2b). The observed profiles clustered along the *η*_s_ − *η*_i_ axis (Fig. 2a), a pattern consistent with the finding that these networks evolve by accumulating and recombining largely intact biochemical modules, each with its own source control (a dietary carotenoid)^33^, with the cohesion among modules maintained by derived carotenoids shared between modules, that necessarily form branching points requiring internal controls (Fig. 1b, category 4). This prevalent mode of avian network evolution might also explain the rarity of peripheral branching requiring sink controls (Fig. 1b) that would be formed by the terminal attachment of nodes to linear enzymatic pathways—a pattern that is rare in birds (Fig. 3)^34^.

### Transfer of controls and its evolutionary consequences

How can a trait as fundamentally dependent on external inputs as diet-derived carotenoid ornamentation acquire internal control? This occurs when increasing redundancy in dietary inputs makes metabolic expansion points more influential in controlling the network^20^—a condition exemplified by network degeneracy^35^, in which structurally distinct biochemical pathways produce an identical end product (Fig. 1a, Supplementary Fig. 5). Specifically, degeneracy of directed networks enables transference of control from dietary inputs to the points of branching of derived products when the outgoing enzymatic connectivity of the derived product exceeds the diversity of dietary pathways that produce it (i.e., when an increase in Ns leads to a decrease in *η*_s_, Fig. 1a)^36^. In avian evolution, this occurs when structurally distinct biochemical modules, each with its own source control and unique derived products, merge at intermediate nodes^33^, necessarily generating the need for additional (internal) control of resulting branching (Fig. 1a). The evolutionary importance of this scenario is that internal controls enable exploration of enzymatic networks, while redundant source controls sustain this exploration by shielding it from environmental fluctuations (Fig. 3a). It follows that frequent addition of modules that share upstream nodes (closer to dietary carotenoids, smaller *L* in Figs. 1a, 3b, c) increases *η*_i_ even in networks fully dependent on external inputs, as was indeed the case in avian lineages under this study (Supplementary Fig. 3b).

Applied to the avian evolution of carotenoid networks, this scenario makes two predictions. First, if evolutionary exploration of enzymatic pathways is sustained by redundancy in source controls (Fig. 3), then the control profiles should change with network growth—the controls should not accumulate as a network grows, but instead accumulation of one type of control should lead to the transference of control to another control type and different nodes becoming controlling as networks grow. Alternatively, simple elongation of existing, source-controlled, biochemical pathways would maintain the same control profiles and nodes over a vast range of network sizes. Second, the network pathways that combine redundancy in dietary inputs with enzymatic expansion of derived compounds (i.e., those with internal control, Fig. 1b, category 4) should be associated with evolutionary diversification. This is expected because these pathways combine a diversity of potential outputs—facilitating both diversification and links between diet-specific adaptations (Fig. 1b)—with greater evolutionary stability of pathways that produce them (Fig. 3).

**Fig. 4:**
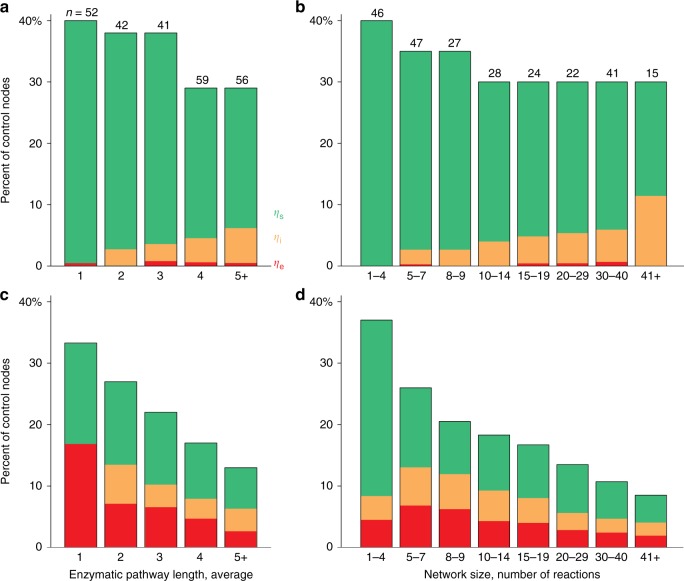
Control profiles change with network size in observed avian networks, but not in their randomized counterparts. **a**, **b** Growth of avian networks does not require changes in proportion of controlling nodes (Nc/N). Instead different nodes acquire control status as a network grows, leading to changes in control profiles. **c**, **d** Randomized counterparts of observed avian networks. ER randomization reveals that growth of avian networks avoids elongation of existing biochemical pathways—producing a sharply decreasing proportion of Nc and the preservation of control profiles across a ten-fold increase in network sizes. Enzymatic pathway length (in reactions) is the longest minimal pathway between a dietary carotenoid and a terminal derived carotenoid deposited in plumage, averaged across pathways. Number of species in each group is given above bars

We found strong support for both predictions. First, across a ten-fold increase in network size and complexity, the proportion of nodes needed to fully control avian networks was strikingly similar, yet the control profiles differed—elongation of enzymatic pathways and greater complexity of networks were accompanied by diversification of control profiles and, specifically, by an increasing proportion of internal controls (Fig. 4a, b). On the contrary, growth of randomized networks led to a progressively declining number of required controls whilst preserving their initial control profiles (Fig. 4c, d). Both observed and randomized networks had a similarly high (30–40%) proportion of control nodes at small sizes. However, larger randomized networks did not disproportionally accumulate internal branching, in contrast to their real-life counterparts (Fig. 4). Because avian carotenoid networks must grow from dietary carotenoids, gains of redundant dietary carotenoids and network cohesion necessitates accumulation of internal controls of enzymatic branching that form when the biochemical modules merge (Fig. 1b). As a result, avian networks are expected to harbor greater potential for innovation and diversification than is produced by randomized network growth, because different combinations of internal controls result in different evolutionary trajectories.

**Fig. 5:**
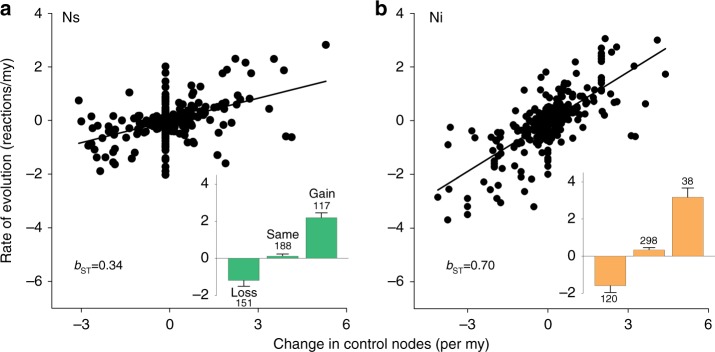
Internalization of control over external inputs enables accelerated occupancy of enzymatic network and faster diversification of carotenoid pathways. Relative contribution of a change in **a** number of source controls, Ns, and **b** number of internal controls, Ni, in extant and ancestral networks to the rate of evolution (reactions/my) of carotenoid pathways. *P* < 0.05 for both standardized regression coefficients (*b*_ST_). Insets show mean ± s.e.m. evolutionary rates (nodes/my) for loss, retention, and gain of control nodes, correspondingly

Second, although the enzymatic branching that required internal controls was encountered in only 8.3% (38/456) of all evolutionary transitions in control profiles (Supplementary Data 2), it accounted for nearly 70% of variation in evolutionary rates of carotenoid pathways (Fig. 5b). This was more than twice the variation than was explained by the thrice more frequent changes in the source controls (117/456 transitions, 26%; Fig. 5a). Similarly, elongation of carotenoid pathways most closely covaried with accumulation of internal controls, and only secondarily with accumulation of source controls (Supplementary Fig. 3a). Averaged across the entire avian tree, a new internal control was gained every 7.14 million years (my) and lost every 4.34 my, whereas a new source control was acquired every 3.12 my and lost every 2.7 my (Supplementary Data 2).

### Evolutionary cycles of control profiles

Two broad patterns can alter the control profile of carotenoid networks: (1) a change in distribution and directionality of reactions, and (2) gain or loss of the control nodes themselves, such as when new dietary inputs are acquired or lost. To determine the relative importance of these patterns, we undertook three further analyses. First, we traced evolutionary trajectories of control profiles of reconstructed ancestral networks across the last 45 my of avian evolution (Supplementary Fig. 4 and Supplementary Data 1). Second, we calculated the rates and likelihoods of historical transitions between the networks of different control profiles (Fig. 1b), and examined correlates of these transitions. Third, we took advantage of the widespread bi-directionality of enzymatic reactions between derived carotenoids (Supplementary Fig. 5 and Supplementary Data 3) and examined the extent to which changes in edge directionality affected control profiles.

**Fig. 6:**
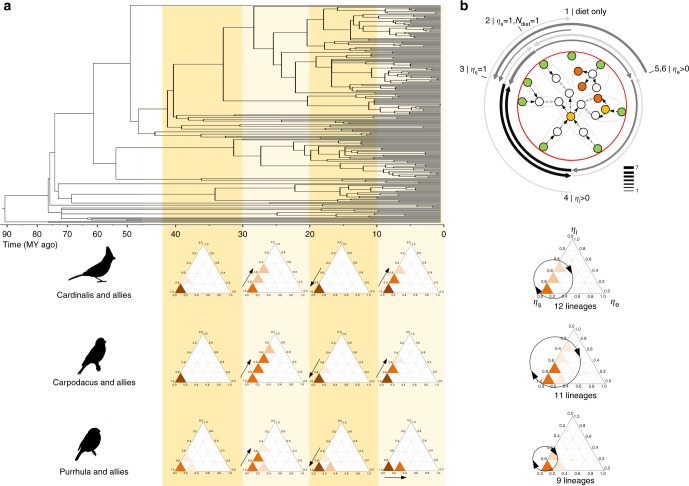
Evolutionary cycles of control profiles. **a** Examples of control profiles in reconstructed networks in three avian groups, taking into account the shared ancestry of the lineages as delineated by phylogeny (Supplementary Fig. 4), binned by 10 my (shaded areas on phylogenetic tree) over the last 45 my. Control profiles of extant species of each group are shown on the right. In all lineages, gains of dietary carotenoids and associated increase in Ns has led to greater redundancy in source controls and, correspondingly lower *η*_s_ and higher *η*_i_ or *η*_e_ in a subsequent period, followed by loss of some dietary carotenoids (Fig. 3, Supplementary Fig. 7) and repeat of the cycle. **b** The basis for the cycles in **a** is equally frequent transition between degenerative, source-dominated networks (category 3 in Fig. 1b) and networks with internal controls (category 4 in Fig. 1b). This transition was closely associated with the gain and loss of additional dietary carotenoids (Supplementary Fig. 7). Shown are the rates (line thickness) and likelihoods (shade) of evolutionary transitions between control categories. Black lines indicate highly likely transitions (zero-value rate parameters (*z*) in less than 1.5% of models), dark gray lines show likely transitions (1.5 < *z* ≤ 10% of models), and light gray shows probable transitions (10 < *z* *<* 20% of models), transitions with *z* > 20% are not shown. Supplementary Fig. 6 shows all results and Supplementary Fig. 1 lists sources for drawings. Cardinalis and allies include genera Cardinalis, Pheucticus, Piranga, Nesospiza, Paroaria, Coereba, Ramphocelus, and Sicalis; Carpodacus and allies include genera Carpodacus, Haematospiza, Uragus, and Euphonia; Pyrrhula and allies include genera Pyrrhula, Rhynchostruthus, Vestiaria, Mycerobas, Coccothraustes, Bucanetes, and Pinicola

We found that transitions between the source-dominated and internal control-dominated networks were cyclical (Fig. 6b and Supplementary Fig. 6), and closely followed gains and losses of dietary inputs (Supplementary Fig. 7). Changes in directionality of enzymatic reactions did not alter network control profiles (Supplementary Fig. 8), whereas gains of redundant dietary carotenoids consistently facilitated acquisition of internal controls (Supplementary Fig. 3b). These patterns were also evident in historical cycles of control profiles within individual lineages (Fig. 6a, Supplementary Fig. 9). For example, in the lineages leading to extant *Carpodacus* species, greater accumulation of source controls at the 40–30 mya period has led to an increase in internal controls up to 70% during the subsequent period (30–20 mya), followed by reduction in *η*_i_ and increase in *η*_s_ associated with loss of some dietary carotenoids. In lineages leading to extant *Pyrrhula* species, the cycles were smaller (up to 50% in *η*_i_ and 20% in *η*_e_) and also involved increases in the sink controls (see also Ploceidae and Estrildidae in Supplementary Fig. 1). The control profile cycle (range of change along *η*_s_ *–* *η*_*i*_ axis per unit of time; Fig. 6a, summary) was proportional to the redundancy of source controls during the cycle (Supplementary Fig. 3b): the acquisition of more dietary inputs enabled greater changes in a network control profile (Supplementary Fig. 7).

## Discussion

Taken together, the results of this study reveal three broad scenarios by which changes in control of avian carotenoid networks affect the tempo and mode of their evolution. One, internalization of network controls, enabled by both gain of redundant dietary inputs (Supplementary Fig. 7) and underlying branching of a biochemical network in the vicinity of the inputs (Fig. 3a), leads to exceptional evolutionary diversification (Fig. 5b, path 3 → 4 in Fig. 6b). Two, gain of dietary inputs without associated internalization of controls (i.e., when network remains source-controlled, but with increasingly redundant sources) produces either stasis or limited elongation of biochemical pathways (Figs. 3, 5a; Supplementary Fig. 7). Three, loss of dietary carotenoids associated with the loss of their redundancy leads to contraction of enzymatic networks to the immediate biochemical vicinity of dietary carotenoids (path 4→1 in Fig. 6b, Supplementary Fig. 6). The likelihood of these scenarios depends on juxtaposition of a species’ diversity of dietary carotenoids and their environmental variability, and distribution of underlying network connectivity^37^. The main contribution of this study is to show that transition in network controllability may be a general mechanistic principle by which these three common scenarios form evolutionary cycles. It further provides a shared mechanistic basis for frequently reported highly variable rates of evolutionary diversification of carotenoid networks across ecological clades of birds, recurrent convergence of these networks between historically and ecologically distant taxa, and the boom-and-bust evolutionary cycles in avian carotenoid networks caused by biochemical redundancy of dietary inputs^37^.

More generally, these findings illuminate a mechanistic basis for the long-standing idea that gains and losses of external dependencies among species drive their evolution^1,3,30^ and show how such dependencies affect the speed of their adaptation^5^. Further, these results support the prediction that the oft documented scaling among categories of controlling structures in biological networks^38^ might be a product of the evolutionary dynamics of the costs and benefits of types of controls^29,39^. Degeneracy of complex biological structures is thought to be an inescapable product of evolution by natural selection^35^, arising as a resolution of competing demands of robustness, needed for local adaptation, and evolvability, required for continuous evolution^8,11,21,24,40^. In this process, current functions elevate some of the existing network elements to the transient roles of network controls, enabling a largely unchanging ancestral structure to nevertheless produce transient adaptive innovations. The continuous evolution of external input-dependent carotenoid phenotypes^41^ might be a particularly tractable example of this general process.

## Methods

### Structural controllability and control profiles

Network controllability measures the ease and ways in which a network can be controlled—such as when a network needs to be guided from one state into another or when information, signals, or materials need to be efficiently propagated through it^19,42^. The nodes that are influential in these processes, either because of their topological position or connectivity are control nodes^20,43^. Here we specifically focused on structural controllability^44^—the changes in network topology and wiring associated with change from one functional network state to another. The use of structural controllability to measure network behavior rests on the assumption that the network structure reflects its internal dynamics^20,27,45^. This assumption makes it particularly appropriate metric for external input driven, directed networks, such as enzymatic networks dependent on dietary carotenoids. Further, in the empirical study of the correspondence between flux and structure in avian carotenoid networks this assumption was upheld^46^.

We were specifically interested in two aspects of the structural controllability: the minimum number of network nodes (Nc) needed for full control of a network and its change^20^—i.e., what portion of nodes are controlling, and the functional associations of these control nodes^29^—that is, why a particular element of a network is controlling in a given network state.

Computationally, Nc is the maximum set of edges (here enzymatic reactions) that do not share either start or end nodes (here carotenoid compounds) and are thus unmatched and need additional controls:^19^ essentially, a control node is not pointed to by any matched edge. In biological networks, the unmatched nodes are those that are not directly reachable from input signals, either because these nodes are isolated from the rest of the network (can only be dietary carotenoids in avian networks) or because these nodes are parts of branching that require additional controls (e.g., either left or right red node on Fig. 1a needs an additional control). By definition, pathway branching is formed by network enzymatic expansion when paths from fewer nodes reach more nodes, i.e., when the upstream nodes at the start of paths are no longer sufficient to fully control downstream nodes.

Because controllability is mechanistically determined by how many neighbors each node can directly control, the degree asymmetry—the difference in number of incoming and outgoing edges—contributes the most to the node’s control propensity^19,20,47^. This asymmetry enables classification of the control nodes into three general classes^29^. Control nodes with in-degree = 0 are source controls (Ns), the control nodes where neither in-degree nor out-degree is zero, but in-degree < out-degree are internal controls (Ni), and control nodes with out-degree = 0 are sink controls (Ne) (Fig. 1a).

We calculated Nc for 467 networks (*n* = 250 extant species and 217 ancestral networks, see below, Supplemental Data 1) using the maximum matching Hopcroft-Karp algorithm^48,49^ with Enthought Canopy and Python 2.7 (code in http://github.com/gusy/lightnx-controllability) and NetworkX 1.9^50^. The algorithm outputs the set of a network’s nodes that are controlled (matched) by an adjacent node (*N*_matched_) in each network configuration. The difference between the network’s total number of nodes (*N*) and *N*_matched_ is the number of nodes needing independent control (*N*_c_). The number of internal controls can be obtained as Ni = Nc-(Ns + Ne)^29^. Standardization of the number of controls of each class by the total number of network controls (Nc) is the control profile of a network, consisting of ratios where *η*_s_ = Ns/Nc, *η*_i_ = Ni/Nc, and *η*_e_ = Ne/Nc (Fig. 1a).

Changes in the direction of reactions can modify degree asymmetry across a network, commonly leading to changes in both the extent of controllability and distribution of control nodes^20,45,51,52^. When reaction directionality varies between configurations of the same network, the reactions can be classified as always, sometimes, or never contributing to the node controllability (also termed critical, redundant, and ordinary edges, correspondingly)^20,53^. We capitalized on a widespread bidirectionality of enzymatic reactions between derived carotenoids in avian carotenoid network (Supplementary Fig. 5), and when a species network encompassed one or more bidirectional reactions, as was the case for 114 species, we calculated controllability statistics for all versions of the network that differed in reaction directionality (Supplementary Data 3). Differences in controllability profiles among network versions are given in Supplementary Fig. 8.

### Phylogeny construction

We constructed an ultrametric 50% majority-rule consensus tree (Supplementary Fig. 4)^34^ from 1000 ultrametric trees (Supplementary Data 4) randomly sampled from the Bayesian pseudo-posterior distribution of the Stage2 MayrAll Hackett source tree from birdtree.org^54,55^ using SumTrees 4.1.0^56^ in DendroPy 4.1.0^57^. To build an ultrametric consensus tree, we included a sample size of 1000 trees from the birdtree.org source tree distribution to allow for reliable parameter estimates^58^. Seven out of the 250 study species were not included in the phylogeny: *Colaptes auratus cafer*—because it was not in the birdtree.org taxa, and six Galliformes species (*Alectoris rufa*, *Gallus gallus domesticus, Meleagris gallopavo, Perdix perdix, Phasianus colchicus*, and *Tetrao urogallus*)—because of the discrepancies with the branch lengths of these taxa with respect to the branch lengths of the rest of the extant species^34^. For Bayesian analyses that required a distribution of trees, a random sample of 1000 ultrametric trees was downloaded from the Bayesian pseudo-posterior distribution of the Stage2 MayrAll Hackett source tree from birdtree.org (Supplementary Data 5).

### Ancestral network reconstruction

Ancestral network reconstruction in an explicitly phylogenetic context requires evaluations of structural relationships and biochemical properties by which the compounds and enzymes are linked in the networks of closely-related species and reconstruction of ancestral networks by either parsimony^5,59,60^ or maximum likelihood approaches^61–63^. Evolutionary changes in the structure of networks, specifically in terms of the gain or loss of reactions and compounds, has been demonstrated empirically in comparisons between known ancestral and extant species^64^ and in directed evolution of metabolic pathways^65,66^. A previous approach to reconstruction of Bayesian networks^67^ infers the most likely evolutionary scenario for each metabolic reaction present in the extant species, but only tracks the gain and loss of reactions and assumes a fixed number of network compounds. To overcome this limitation, we used a modified maximum likelihood approach^5^ to test the fit of models of network evolution in the phylogeny and to track ancestral states of compounds and reactions simultaneously^33,37^.

Briefly, we used the ultrametric consensus tree (Supplementary Data 4) in individual, joint maximum-likelihood estimations for ancestral state reconstructions of each of the *n* = 55 carotenoids and 88 enzymatic reactions that occurred at least once in the study species’ networks (Supplementary Data 1). The compounds and reactions were considered discrete (present or absent) and unordered traits, and we tested two Markov models of binary trait evolution in *r8s* (version 1.8)^68,69^ for each compound and reaction: the *Binary-1* model was a one parameter model that assigns equal rates of the gain and loss of a trait and the *Binary-2* model was a two parameter model that allows for different rates of trait gain and loss across the phylogeny. In each Markov model, the reconstructed ancestral state (present or absent) of a compound or a reaction was assigned to each of the 217 internal nodes in the phylogeny by *r8s*, and the ancestral states and rates of gain and loss in the model with the lower Akaike information criterion (AIC) value were retained (Supplementary Data 2). The ancestral network for each internal node in the phylogeny comprised all of the compounds and reactions that were present at the node in the ancestral reconstructions^33^ and is given in Supplementary Data 1. All published data^34,37^ on avian carotenoid networks were assembled according to the protocol^37^.

### Network randomization

Ten random networks were generated for each species and ancestral network according to the Erdös–Rényi (ER) model^70^ using the package igraph 1.0.1^71^ in R 3.3.3^72^. An example of R code model for network randomization is given in Supplementary Note 1 and illustrated in Supplementary Fig. 10. For a network comprised of *n* compounds and *r* reactions, a random network was generated by adding directed reactions between pairs of *n* compounds uniformly randomly from the set of all possible directed reactions between all *n* compounds until *r* reactions were present in the network. Self-loops representing a reaction between a compound and itself were not allowed. This randomization procedure thus generated networks that always had the same number of compounds and reactions as the species or ancestral networks, but the degree distribution of each random network varied in relation to the real network. Both the number and direction of the reactions connected to each compound in the random networks therefore varied across each of the random networks (Supplementary Fig. 10). This was crucial for the analysis because both the number of reactions and their directionality affect the control properties of compounds^29,36^.

### Independent contrasts and phylogenetic trajectories

Phylogenetically independent linear contrasts were calculated with the package ape 5.0^73^ in R 3.4.4 based on the ultrametric 50% majority rule consensus tree with randomly resolved polytomies (Supplementary Data 4). For the illustration with three large clades on Fig. 6a, control profiles of the ancestral networks were binned in 10 million year (my) intervals and summary ternary plots for each group were calculated. We limited this analysis to the last 45 million years of avian evolution, because reconstruction of biochemical networks was less reliable at deeper nodes^33^. Supplementary Fig. 9 shows control profiles of reconstructed networks binned in 5 my and 15 my intervals. Bins ≤ 10my were most informative because average gain and loss of internal controls in the studied species typically occurred at intervals shorter than 10 my (Supplementary Data 2), and bins longer than 10 my tended to combine cycles (Supplementary Fig. 9b).

### Compound structural redundancy

The biochemical redundancy is a property of each metabolized carotenoid (also called biochemical degeneracy). It is measured as the total number of pathways with no repeated compounds, known as simple paths, between a metabolized carotenoid and all 14 dietary carotenoids of the combined avian network (Supplementary Fig. 5)^34,37^. All simple paths between a dietary and a derived carotenoid were generated using a modified depth-first search algorithm^74^ in the program NetworkX 2.0^50^ in Python 2.7.13, and are given in Supplementary Data 6.

### Bayesian analyses

To determine the sequence and rates of transitions between the categories of network control configurations (Fig. 6, Supplementary Figs. 6 and 7, Supplementary Data 7), we used the program MULTISTATE^75,76^. We examined correlated evolution between the number of dietary carotenoids and transitions between the categories of network controls with the program DISCRETE^76–78^. We compared the marginal likelihoods, estimated with a stepping stone sampler using a beta distribution with the parameters *α* = 0.4 and *β* = 1.0, and with 100 stones and 1000 iterations each, of models where the traits were assumed to evolve independently or in a correlated manner (Supplementary Note 2). We repeated each analysis three times to confirm stability of the harmonic mean of the likelihoods. For the DISCRETE models, the network control configurations were combined into two classes—*η*_*s*_-dominated (categories 2 and 3 in Fig. 1b) and *η*_*i*_-dominated (categories 4 and 5); category 6 was excluded because of low sample size. The number of dietary carotenoids was classified into two classes—low (1–2 dietary carotenoids) and high (>2) (Supplementary Fig. 7, Supplementary Data 8 and 9). Coevolution of control profile transitions and dietary inputs was also analyzed with regressions on phylogenetic independent linear contrasts (Supplementary Fig. 3).

For both MULTISTATE and DISCRETE analyses, we used reverse-jump Markov chain Monte Carlo^75–78^ with a 1000-tree distribution^54^. This method accounts for uncertainty in phylogenetic relationships and visits evolutionary model and parameter estimate combinations in proportion to their posterior probabilities. To facilitate estimation of evolutionary transition rates the trees were scaled to have a mean branch length of 0.1^76^. We ran the Markov chain for 41 million iterations with a one-million iteration burnin. The chain was sampled every 20,000 iterations. To explore the influence of the priors on estimated evolutionary transition rates, we used both uniform priors drawn from a distribution of 0–100 and a hyper-prior approach where a uniform distribution of 0–100 was used to seed an exponential prior (Supplementary Note 2). The results for both sets of priors were similar, thus we report only evolutionary transition values derived from the hyper-prior approach.

### Reporting summary

Further information on experimental design is available in the Nature Research Reporting Summary linked to this article.

## Supplementary information

Supplementary Information

Reporting Summary

Description of Additional Supplementary Files

Supplementary Data 1

Supplementary Data 2

Supplementary Data 3

Supplementary Data 4

Supplementary Data 5

Supplementary Data 6

Supplementary Data 7

Supplementary Data 8

Supplementary Data 9

## Data Availability

All data are available in the manuscript and the supplementary materials.
