# Supplementary material for: Cycles of external dependency drive evolution of avian carotenoid networks
Source: Nat Commun. 2019 Apr 8;10:1596. doi: 10.1038/s41467-019-09579-y (PMC6453931; doi:10.1038/s41467-019-09579-y)
Supplement: Supplementary file 1 — Supplementary Information [file 41467_2019_9579_MOESM1_ESM.pdf]

Supplementary Information

Cycles of external dependency drive evolution of avian carotenoid networks

Badyaev et al.

Supplementary Figures:

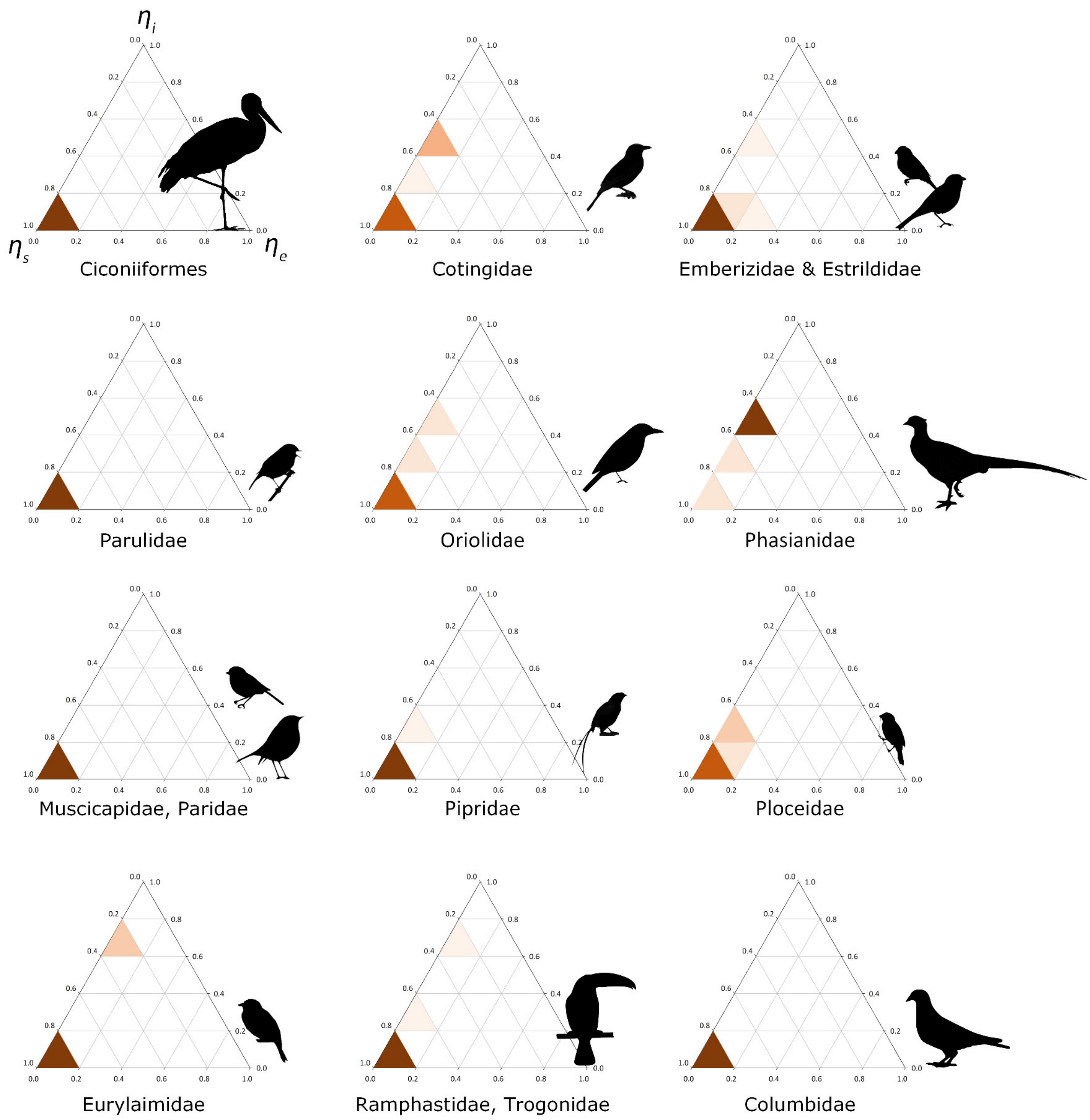

Supplementary Figure 1. Clustering of control profiles of avian carotenoid networks. Shades indicate the density of networks (in 20% increments) that have control profiles in shaded ternary space. Bird drawings, silhouettes, and photos are by the authors or in public domain as follows: Oriolidae is from http://phylopic.org/image/f3c3d9a5-e4d6-48da-9641-aecff91c4b8c/ (by L. Shyamal); Emberizidae is from http://phylopic.org/image/42fdc3cb-37fc-4340-bdf9-eed8e050137c/ (by L. Shyamal), Estrildidae is from http://phylopic.org/image/fb67708c-039c-4beb-9e0e-421c5c5e4b5a/ (by T. M. Keesey), and Paridae is from http://phylopic.org/image/dfdfb59e-8126-44e1-a7a9-1bf698113e1c/.

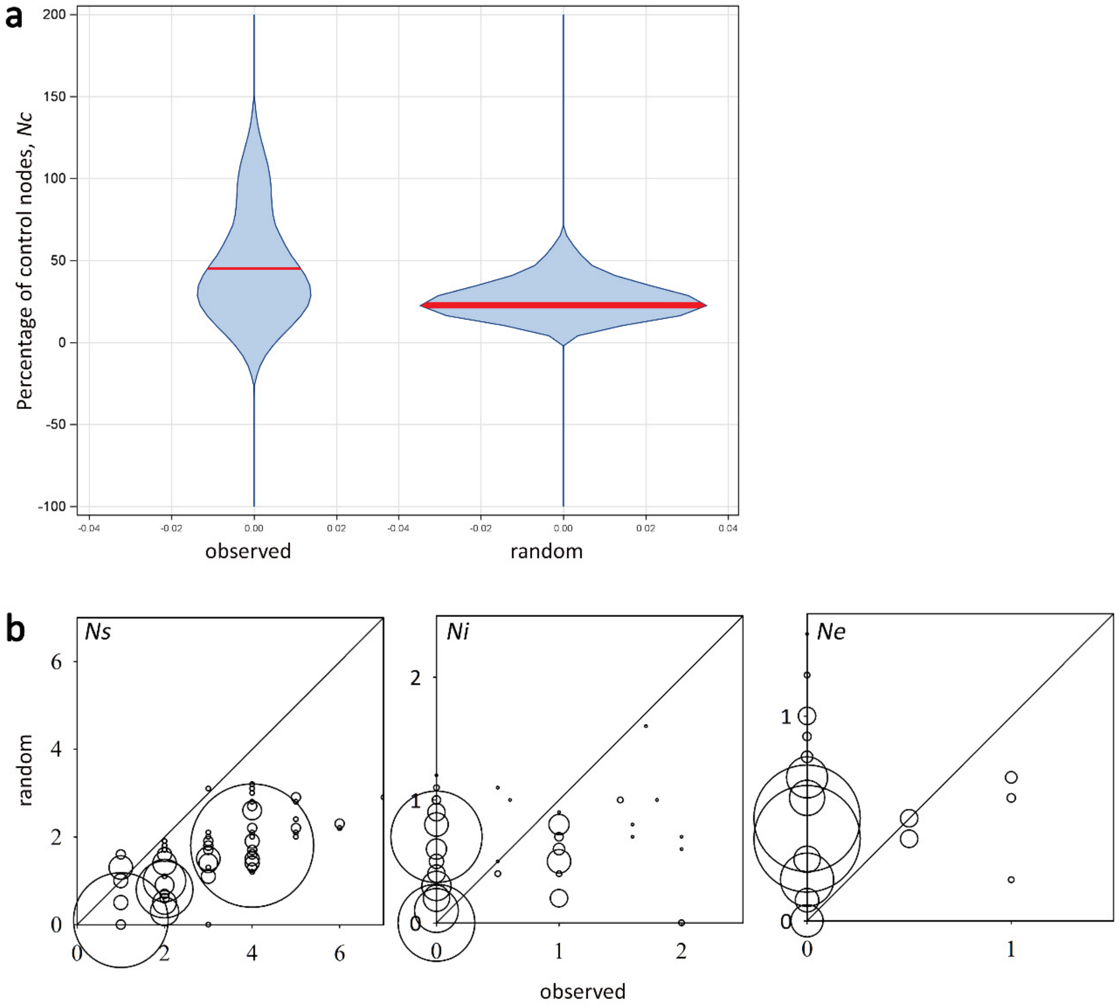

Supplementary Figure 2. Differences in controllability between observed avian networks and their randomized counterparts. (**a**) Real-life networks require more of their nodes to be controlled than randomized networks of the same size and complexity. Red band shows mean ± s.e.m. **(b)** Observed networks have more source controls (Ns), but fewer internal (Ni) and sink (Ne) controls than their randomized counterparts. Size of bubbles is the number of overlapping data points. Points on diagonals would indicate no difference between observed and randomized networks.

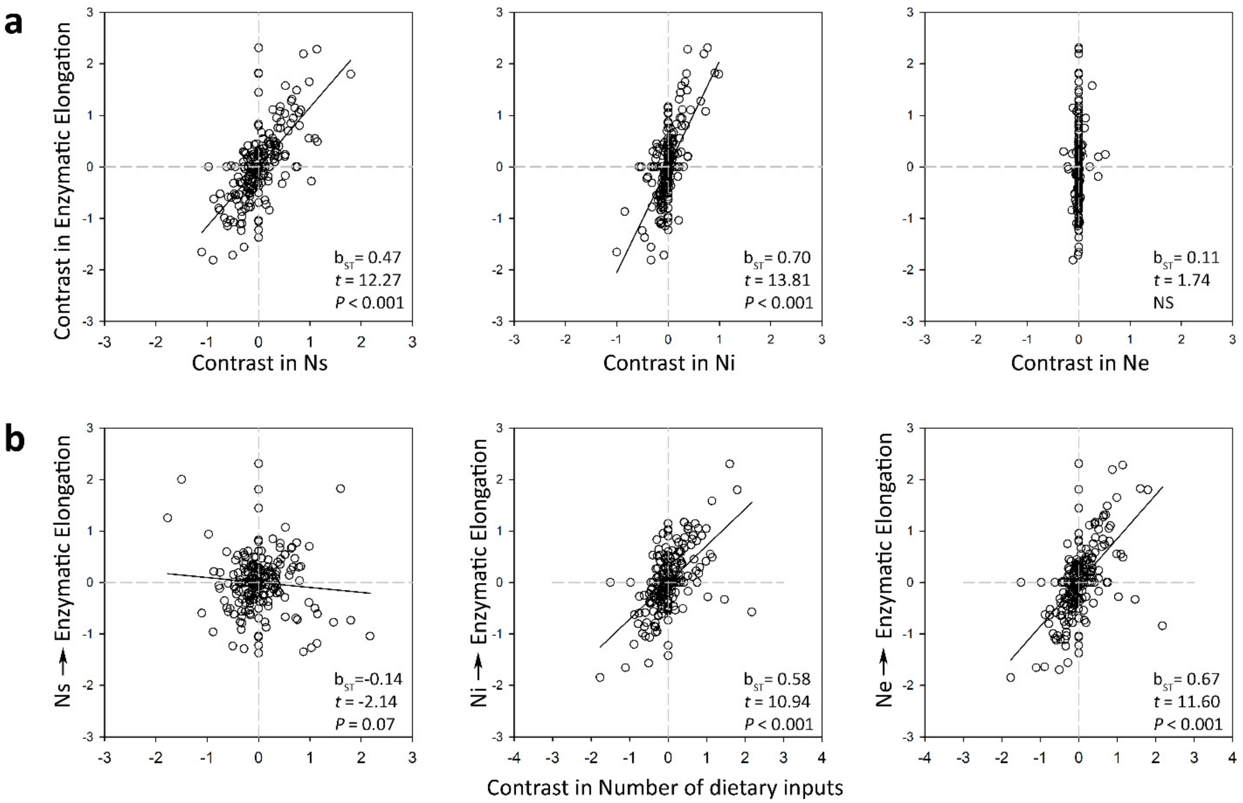

Supplementary Figure 3. Gains of dietary carotenoids and associated redundancy of source controls facilitate internalization of controls, leading to faster network growth. (**a**) Evolutionary elongation of enzymatic pathways (distance from dietary carotenoids to the most derived carotenoid) is most closely associated with gain of internal controls (Ni) followed by gain of source controls (Ns). Gains in sink controls (Ne) occur rarely in birds (Fig. 2a) and are not associated with enzymatic elongation. (**b**) The effect of change in the number of dietary inputs on the effect of controls on enzymatic elongation shown above. In the Ns case, gains of dietary entries marginally weaken the effects, in the Ni and Ne cases, gains of dietary entries significantly strengthen the effect. All partial regression plots are from the same model based on phylogenetic independent linear contrasts. All regressions are through the origin.

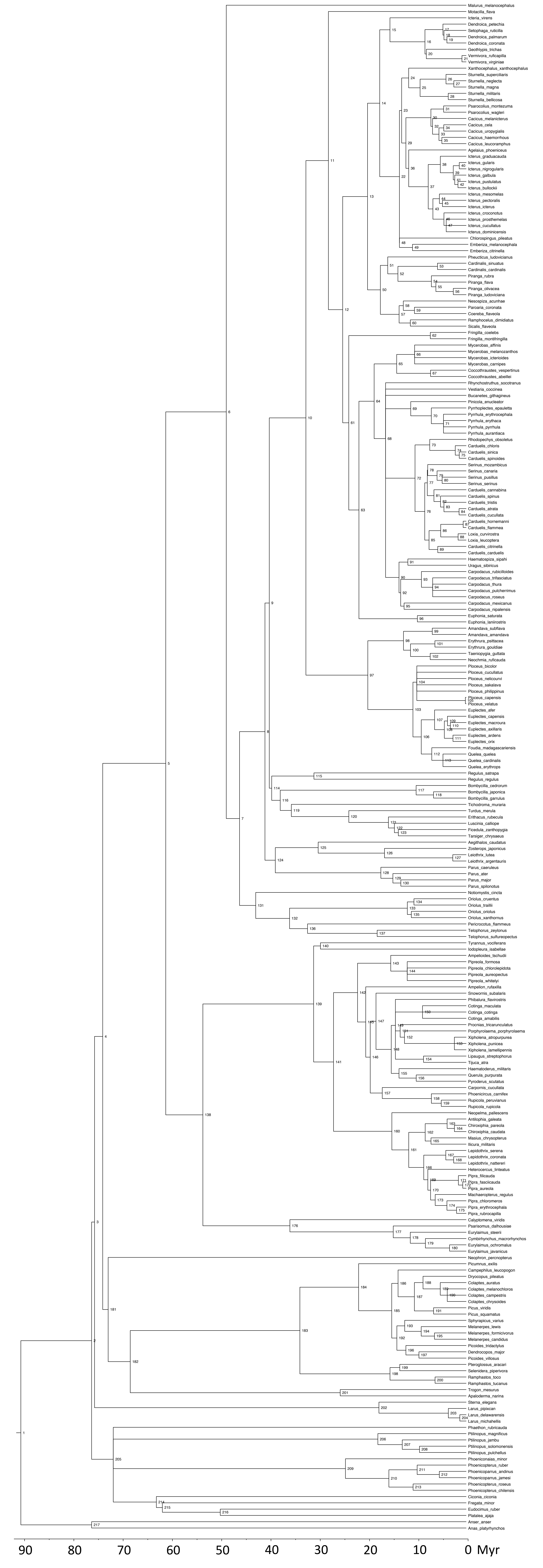

Supplementary Figure 4. Majority-rule ultrametric consensus tree for species in this study showing states of reconstructed ancestral networks at internal nodes (numbers refer to networks shown in Supplementary Data 2).

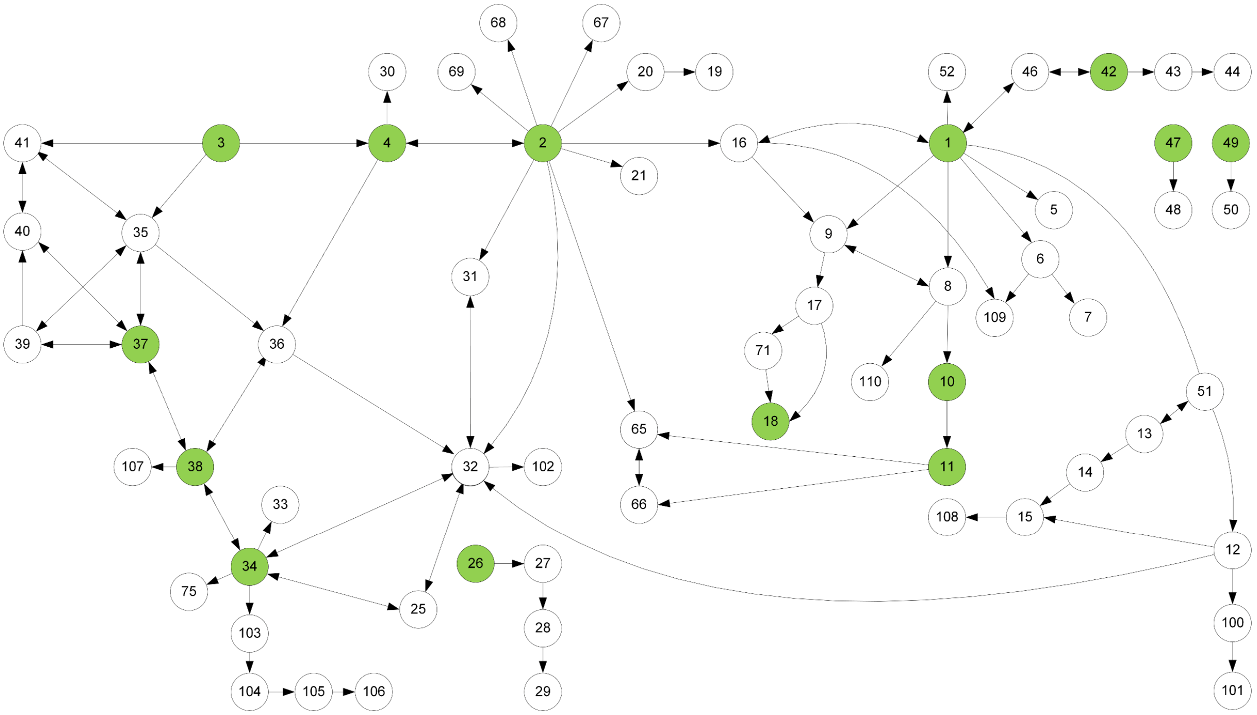

Supplementary Figure 5. Directionality of enzymatic pathways linking carotenoids that occur in species under this study. Numbers in nodes correspond to carotenoid names listed in Supplementary Data 2. Green nodes are carotenoids that are known to be dietary in some species. Note that a dietary carotenoid in one species' network can be a derived carotenoid in another and thus directionality of a reaction often varies between species even for dietary carotenoids. The schematics is the summary of all published carotenoid pathways[34, 37]. The network shows a number of bidirectional reactions (double-headed arrows). When a species network encompasses such bidirectional pathways (Supplementary Data 3), the network's structural controllability (Nc, Ns, Ni, and Ne) was calculated for network configurations with reactions in both directions.

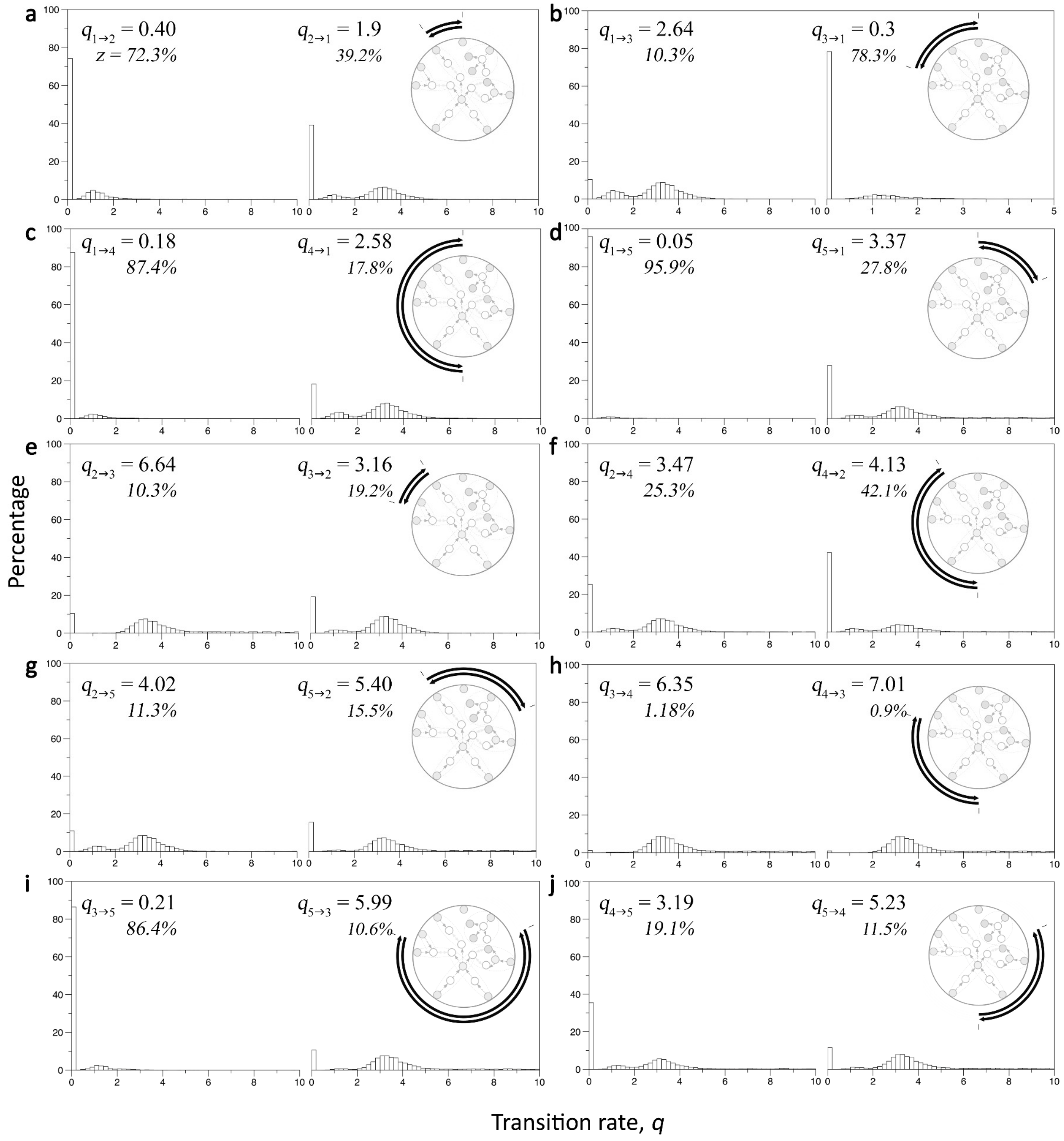

Supplementary Figure 6. The rates and likelihoods of evolutionary transitions between the network categories with different control profiles (Fig. 1b). External control categories 5 and 6 are combined. Shown are the transition rate (q) histograms estimated with rj-MCMC (see Methods) with exponential hyper priors and % of models with zero values for a focal direction of transition. Both uniform and hyper priors fit the models equally well (Minimum Bayes Factor = 1.12). Only likely evolutionary transitions ($z < 20\%$) are illustrated in Fig. 6b.

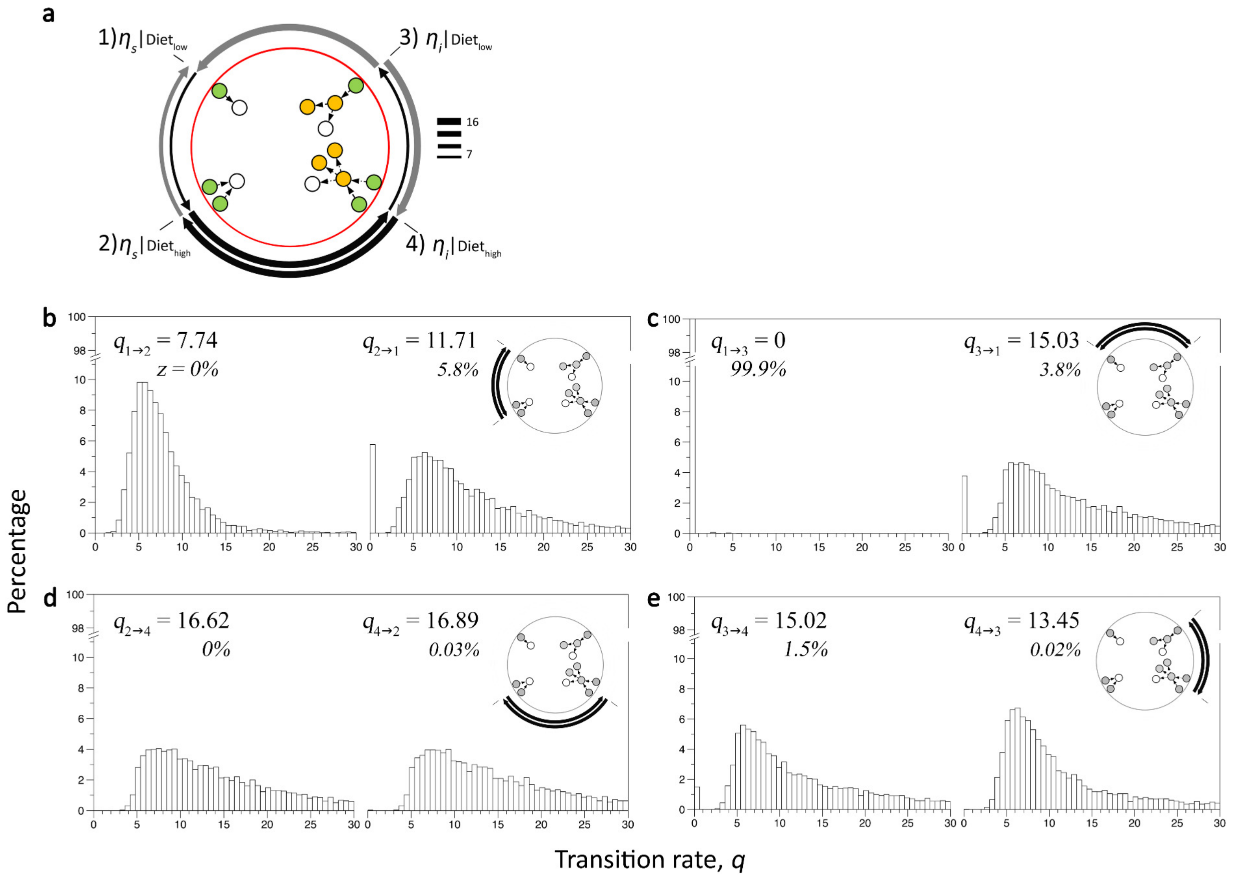

Supplementary Figure 7. Transition from source-controlled networks to internally-controlled networks only occurs after a gain of additional dietary input. (a) Evolutionary transitions between source- and internally controlled networks are closely associated with gain and loss of dietary carotenoids (Minimum Bayes Factor = 33.412). Source-controlled networks with low dietary inputs do not transfer into internal-control state (**c,** 1→3), often gain dietary carotenoids (**b,** 1→2), and are a frequent end state of internally-controlled networks losing dietary carotenoids (**c,** 3→1). In networks supported by several dietary inputs, transitions between source- and internal-control states are frequent (**d,** 2 → 4, 4→2). Shown are the transition rate histograms and averages (*q*) and % of models with zero values for a focal direction of transition. In (a) arrow thickness indicates frequency of transitions (shown in the legend) and black lines indicate highly likely transitions (zero-value in <1 % of models), grey lines show likely transitions (1< z <6% of models).

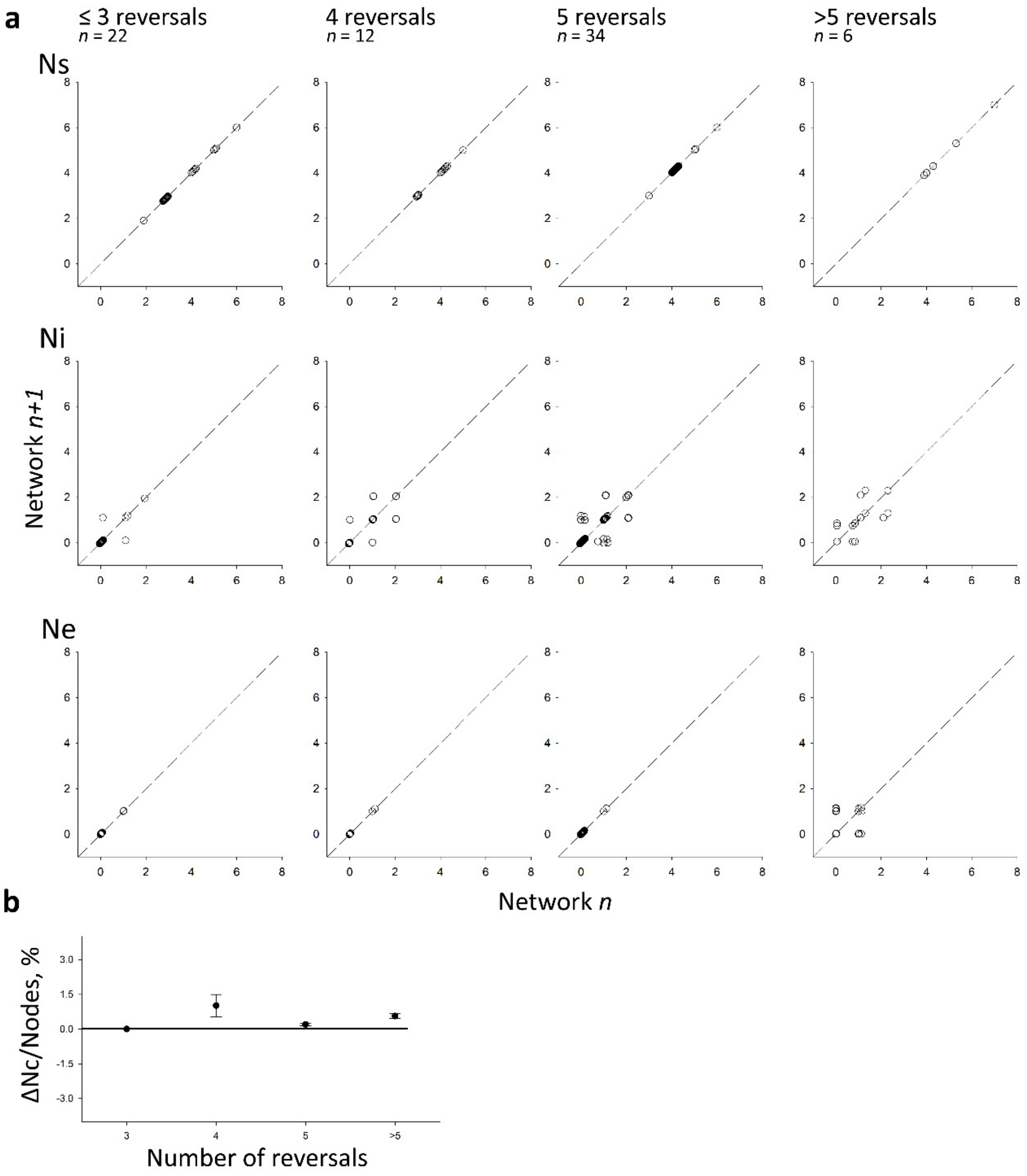

Supplementary Figure 8. Directionality reversals do not significantly alter distribution of degree asymmetry across configurations of the same network, and have minimal effect on network controllability. Shown are pairwise comparisons of (**a**) Ns, Ni and Ne between networks of the same species but with reversed directionality of reactions (graphs are arranged by columns with the number of reversals within a network, Supplementary Data 3). Points on diagonal indicate no difference in the number of control nodes between network versions. (**b**) Change in the percentage of control nodes out of the total number of nodes in a network (mean ± s.e.m.) does not exceed 1.5% even for the maximum number of directionality reversals. Within each species network, only a small portion of reactions can reverse directionality (because dietary carotenoids only have outgoing reactions), explaining why the overall effect of directionality is limited in avian networks.

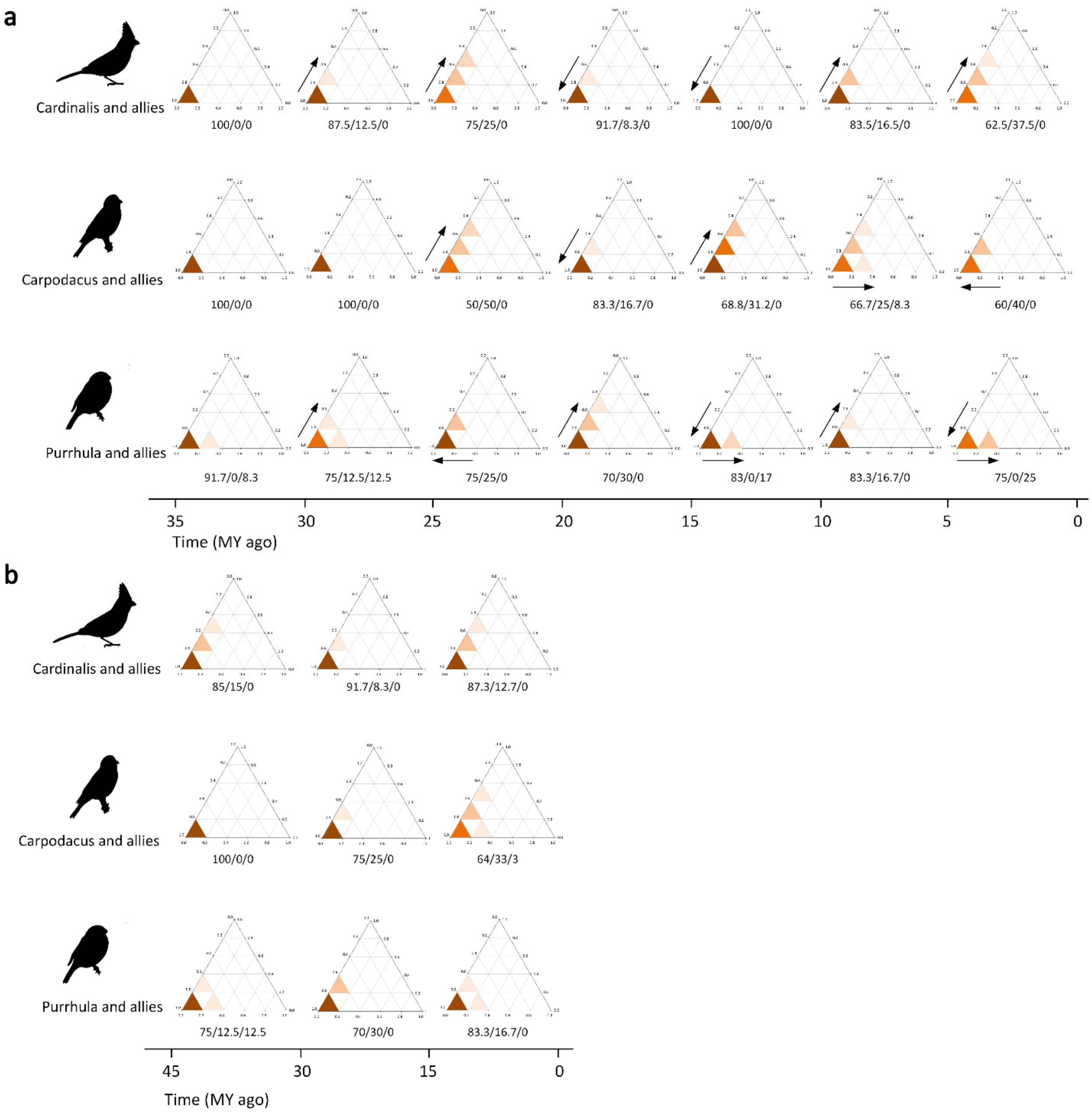

Supplementary Figure 9. Evolutionary transitions in control profiles. Examples of control profiles in reconstructed networks from 0 to 45 my ago in three avian lineages binned by 5 my **(a)** and 15 my **(b)**. Fig. 6 of main text shows bins of 10 my. Numbers under ternary plots are average control profiles ($\eta_S/\eta_i/\eta_e$ ratios) for the entire period (5 or 15 my). The plots show density of networks (in 15% increments) with control profiles in that ternary space. The transference of control profiles (Fig. 6) is most evident in 10 my and 5 my bins, since they best reflect average gain and lost of internal controls (7.14 my and 4.34 my correspondingly, Main text).

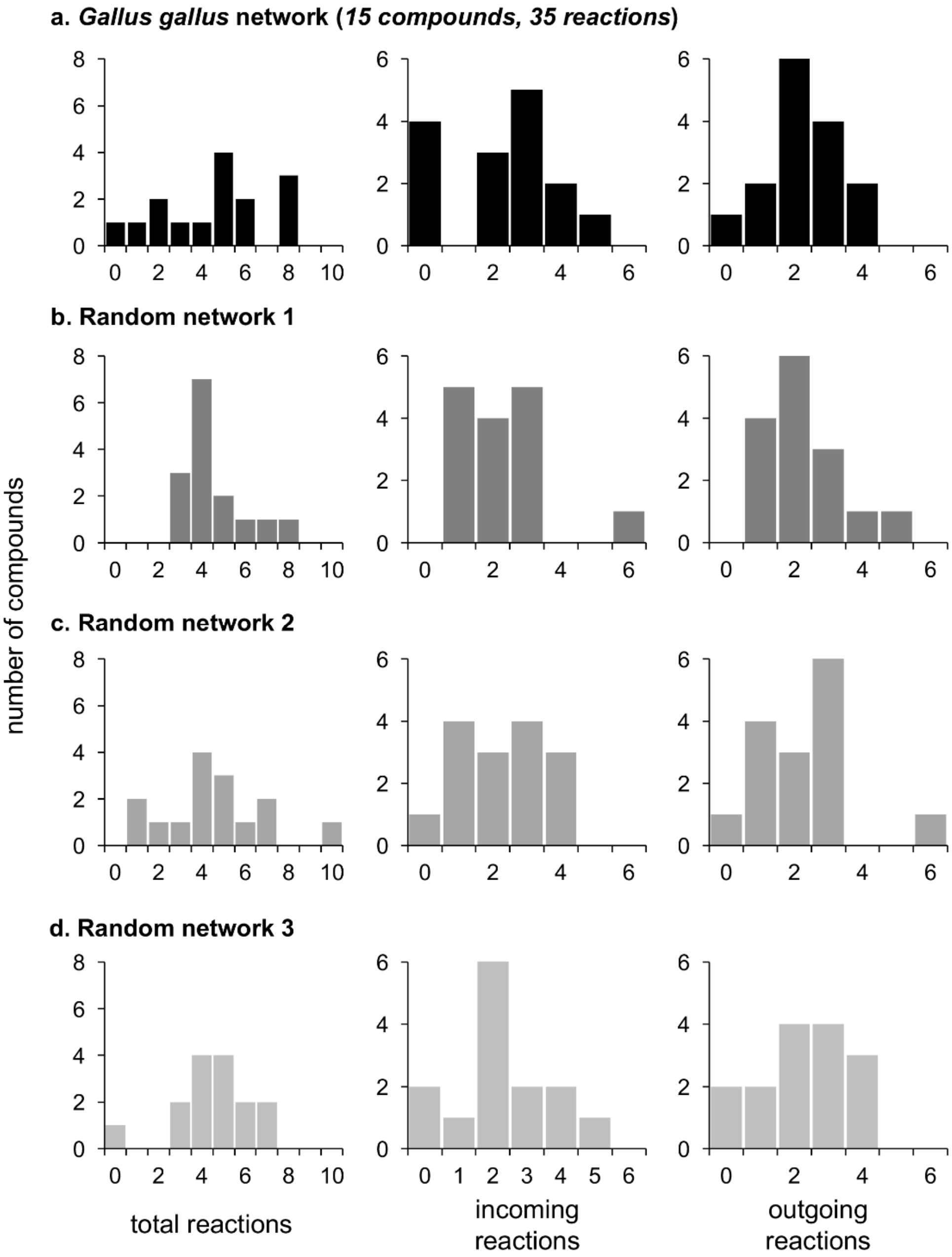

Supplementary Figure 10. An example of a species network and three of its random networks with the same number of compounds and reactions, but variable degree distributions. Distribution of total (first column), incoming (second column), and outgoing (third column) degrees for (**a**) the carotenoid metabolic network of *Gallus gallus*, and (**b-d**) three of the ten random networks generated from the 15 compounds and 35 reactions present in this species. The random networks were generated according to the Erdös–Rényi model, which adds directed reactions between pairs of the 15 compounds uniformly randomly from the set of all possible directed reactions between all 15 compounds until 35 reactions are present in the network. See Supplementary Note 2 and Methods.

<u>Supplementary Note 1:</u> An example of R code for Erdös–Rényi model for network randomization

```
install.packages("igraph")
library(igraph)

#generates random graph with compounds, reactions, gnm = number of reactions that need to be generated with constant probability, directed=directed graph
g <- erdos.renyi.game(18,36,type=c("gnm"),directed = TRUE)

#makes random network into a dataframe bigraph
g1 <- as.data.frame(get.edgelist(g))

#exports random network bigraph file, for mac: "~/../Controllability/16_31_2.txt"
write.table(g1,"C:/../../Controllability/18_36_10.txt", sep="\t")

#vector of the outdegrees of each compound in random network
outdeg <- degree(g,mode=c("out"), loops=FALSE, normalized=FALSE)

#vector of the indegrees of each compound in random network
indeg <- degree(g,mode=c("in"), loops=FALSE, normalized=FALSE)

#calculate number of sources Ns, number of compounds with 0 incoming reactions
sink <- sum(outdeg == 0)
print(source <- sum(indeg == 0))
#calculate number of external controls, when #sinks > #sources
external <- if((sink-source)>0){
  print(sink-source)
} else {
  print(0)
}
```

<u>Supplementary Note 2:</u> Command line files for Bayesian analyses

1) Multistate analyses of Supplementary Data 8 file:

1.1 Multistate_hp_input. Command file to run the multistate analysis with hyper priors

```
==
1
2
scaletrees
stones 100 1000
it 41000000
bi 1000000
sa 20000
rjhp exp 0 100
lf multistate_controlability_Cat5_exp_rep.log.txt
run
==
```

1.2 Multistate_uniform_input. Command file to run the multistate analysis with uniform priors

```
==
1
2
scaletrees
stones 100 1000
it 41000000
bi 1000000
sa 20000
rj uniform 0 100
lf multistate_controlability_Cat5_uniform_rep.log.txt
run
==
```

2) Discrete analyses of Supplementary Data 9 file:

2.1 Discrete_dep_hp_input. Command file for the discrete analysis with the dependent model andhyper priors

==

3

2

scaletrees

stones 100 1000

it 41000000

bi 1000000

sa 20000

rjhp exp 0 100

lf Cat_var_dep_rep.log.txt

run

==

2.2 Discrete_dep_uniform_input. Command file to run the discrete analysis with the dependent model and uniform priors

==

3

2

scaletrees

stones 100 1000

it 41000000

bi 1000000

sa 20000

rj uniform 0 100

lf Cat_var_dep_rep.log.txt

run

==

2.3 Discrete_indy_hp_input. Command file to run the discrete analysis with the independent model and hyper priors

==

2

2

scaletrees

stones 100 1000

it 41000000

bi 1000000

sa 20000

rjhp exp 0 100

lf Cat_var_indy_rep.log.txt

run

==

2.4 Discrete_indy_uniform_input. Command file to run the discrete analysis with the independent model and uniform priors

==

2

2

scaletrees

stones 100 1000

it 41000000

bi 1000000

sa 20000

rj uniform 0 100

lf Cat_var_indy_rep.log.txt

run

==
