# Supplementary material for: Cycles of external dependency drive evolution of avian carotenoid networks
Source: Nat Commun. 2019 Apr 8;10:1596. doi: 10.1038/s41467-019-09579-y (PMC6453931; doi:10.1038/s41467-019-09579-y)
Supplement: Supplementary file 2 — Reporting Summary [file 41467_2019_9579_MOESM2_ESM.pdf]

nature research

Corresponding author(s): NCOMMS-18-32768B

Last updated by author(s): Mar 6, 2019

Reporting Summary

Nature Research wishes to improve the reproducibility of the work that we publish. This form provides structure for consistency and transparency in reporting. For further information on Nature Research policies, see Authors & Referees and the Editorial Policy Checklist.

Statistics

For all statistical analyses, confirm that the following items are present in the figure legend, table legend, main text, or Methods section.

n/a	Confirmed	
☐	☒	The exact sample size (n) for each experimental group/condition, given as a discrete number and unit of measurement
☐	☒	A statement on whether measurements were taken from distinct samples or whether the same sample was measured repeatedly
☐	☒	The statistical test(s) used AND whether they are one- or two-sided<br>*Only common tests should be described solely by name; describe more complex techniques in the Methods section.*
☐	☒	A description of all covariates tested
☐	☒	A description of any assumptions or corrections, such as tests of normality and adjustment for multiple comparisons
☐	☒	A full description of the statistical parameters including central tendency (e.g. means) or other basic estimates (e.g. regression coefficient) AND variation (e.g. standard deviation) or associated estimates of uncertainty (e.g. confidence intervals)
☐	☒	For null hypothesis testing, the test statistic (e.g. F, t, r) with confidence intervals, effect sizes, degrees of freedom and P value noted<br>*Give P values as exact values whenever suitable.*
☐	☒	For Bayesian analysis, information on the choice of priors and Markov chain Monte Carlo settings
☒	☐	For hierarchical and complex designs, identification of the appropriate level for tests and full reporting of outcomes
☒	☐	Estimates of effect sizes (e.g. Cohen's d, Pearson's r), indicating how they were calculated

Our web collection on statistics for biologists contains articles on many of the points above.

Software and code

Policy information about availability of computer code

Data collection	No software was used for data collection
Data analysis	Scripts for controllability analyses are in Github with links included in the text. All other used software (R, Cytoscape, BayesTraits, and SAS) are publicly available with links and references included in the text. All scripts for network randomization and BayesTraits are included in Supplementary Information

For manuscripts utilizing custom algorithms or software that are central to the research but not yet described in published literature, software must be made available to editors/reviewers. We strongly encourage code deposition in a community repository (e.g. GitHub). See the Nature Research guidelines for submitting code & software for further information.

Data

Policy information about availability of data

All manuscripts must include a data availability statement. This statement should provide the following information, where applicable:

- Accession codes, unique identifiers, or web links for publicly available datasets
- A list of figures that have associated raw data
- A description of any restrictions on data availability

All data are available in the Supplementary Materials and Source Data Files 1-6.

Field-specific reporting

Please select the one below that is the best fit for your research. If you are not sure, read the appropriate sections before making your selection.

☐ Life sciences ☐ Behavioural & social sciences ☒ Ecological, evolutionary & environmental sciences

For a reference copy of the document with all sections, see nature.com/documents/nr-reporting-summary-flat.pdf

Ecological, evolutionary & environmental sciences study design

All studies must disclose on these points even when the disclosure is negative.

Study description	We derived and analysed controllability profiles for published metabolic networks, specifically examining covariates of transition between control profile categories, correlates of linear vs degenerative path elaborations, and historical changes in topology of network control.
Research sample	All data published to January 2016 were included in this study
Sampling strategy	We included data from all publications referenced by Web of Science and Google Scholar following previously published protocols, referenced in this study
Data collection	see above
Timing and spatial scale	All data published as of January 2016were included in this work
Data exclusions	No data were excluded. Some species could not be analyzed by phylogenetic independent contrasts because of unresolved phylogeny as mentioned in the Methods.
Reproducibility	All data and associated script needed to reproduce the results are included or referenced in the study
Randomization	We used ER randomization of network to separate geometric and biological aspects of control profile change with network growth. All methods, scripts used in randomization, and justification are included in the study
Blinding	Each coauthor was responsible for their own, independent derivation of distinct set of measures. These are listed in Author Contributions. Metrics were not shared until all the derivations and datasets were completed.

Did the study involve field work? ☐ Yes ☒ No

Reporting for specific materials, systems and methods

We require information from authors about some types of materials, experimental systems and methods used in many studies. Here, indicate whether each material, system or method listed is relevant to your study. If you are not sure if a list item applies to your research, read the appropriate section before selecting a response.

Materials & experimental systems

n/a	Involved in the study	
☒	☐	Antibodies
☒	☐	Eukaryotic cell lines
☒	☐	Palaeontology
☒	☐	Animals and other organisms
☒	☐	Human research participants
☒	☐	Clinical data

Methods

n/a	Involved in the study	
☒	☐	ChIP-seq
☒	☐	Flow cytometry
☒	☐	MRI-based neuroimaging
