# Supplementary material for: Cycles of external dependency drive evolution of avian carotenoid networks
Source: Nat Commun. 2019 Apr 8;10:1596. doi: 10.1038/s41467-019-09579-y (PMC6453931; doi:10.1038/s41467-019-09579-y)
Supplement: Supplementary file 3 — Description of Additional Supplementary Files [file 41467_2019_9579_MOESM3_ESM.pdf]

Description of Additional Supplementary Files

Supplementary Data 1.xlsx: The species' and ancestral networks

Supplementary Data 2.xlsx: Tests of two Markov models of binary trait evolution for the ancestral reconstruction of compounds and reactions in species' networks.

Supplementary Data 3.xlsx: The network versions used in the calculations of controllability profiles in species' networks with bidirectional reactions

Supplementary Data 4.txt: Ultrametric majority rule phylogeny including the resolved polytomy consensus tree and 1000 tree sample used to build tree.

Supplementary Data 5.txt: Tree sample (n=1000) for species used for evolutionary transitions analysis

Supplementary Data 6.xlsx: Degeneracy measures: number and length of paths between compound and all dietary carotenoids

Supplementary Data 7.txt: Input tree file for BayesTraits analyses

Supplementary Data 8.txt: Input data for the multistate analysis with BayesTraits

Supplementary Data 9.txt: Input data for the discrete analyses with BayesTraits
